# Gonorrhoea among China’s aging population: a 20-year nationwide analysis of epidemiological trends with 5-year projections

**DOI:** 10.3389/fpubh.2025.1594289

**Published:** 2025-06-27

**Authors:** Rui-Rui Peng, Zuo-Xi Chen, Juan Wu, Mei Shi, Xin Zheng, Chun-Jie Liao, Lin Zhu, Xiang-Dong Gong, Fu-Quan Long

**Affiliations:** ^1^Department of Sexually Transmitted Disease, Center of Infectious Skin Disease, Shanghai Skin Disease Hospital, School of Medicine, Tongji University, Shanghai, China; ^2^Department of Sexually Transmitted Disease Epidemiology, Institute of Dermatology, Chinese Academy of Medical Sciences and Peking Union Medical College, Nanjing, China

**Keywords:** older adults, gonorrhoea, sexually transmitted infection, incidence rate, long short-term memory model, prediction

## Abstract

**Background:**

Advances in longevity and pharmacological interventions have facilitated sustained sexual activity among older adults, increasing their vulnerability to sexually transmitted infections. Existing research on older adults in China has largely concentrated on HIV and syphilis, leaving critical gaps in knowledge regarding gonorrhoea.

**Objectives:**

We aimed to analyze trends in gonorrhoea incidence among Chinese older adults aged 60 years and above from 2004 to 2023, and to forecast infection trajectories over the next 5 years.

**Methods:**

Data were sourced from the National Center for STD Control and the National Bureau of Statistics Yearbooks, standardized and stratified by gender and age groups. Temporal trend analysis utilized Joinpoint regression, and prediction model was developed utilizing an optimized Long Short-Term Memory model.

**Results:**

We found: (1) An overall declining yet fluctuating incidence rate (AAPC −5.84; 95% CI, −10.13 to −1.34) with three distinct phases; (2) A consistent predominance of cases among males across all age groups; (3) Slower decline rates in older age groups, particularly among those aged ≥80 years; and (4) A significant reduction in incidence rates during the COVID-19 pandemic. We projected stabilization of overall gonorrhoea incidence rates over the next 5 years (APC −3.22; 95% CI, −6.69 to 0.37), with pronounced upward trend anticipated in the ≥80 age group (APC 20.09; 95% CI, 7.71 to 33.89).

**Conclusion:**

The study highlights persistent gonorrhoea transmission risks among older adults in China, particularly the upward trajectory in the ≥80 age group. These findings call for integrating geriatric sexual health education with strengthened monitoring systems to address evolving epidemiological patterns.

## 1 Introduction

The global population aged 60 years and above is expanding at an unprecedented rate, projected to reach 1.4 billion by 2030 and 2.1 billion by 2050, with developing countries experiencing the most accelerated growth (1). This demographic shift coincides with evolving sexual health dynamics (2). Improved longevity and pharmacological advancements enable sustained sexual activity and changing social norms have collectively increased older adults’ vulnerability to sexually transmitted infections (STI) (3). While the Global Burden of Disease Study 2019 reported a 14% decline in age-standardized STI incidence rate among older adults aged 60 to 89 years from 1990 to 2019, absolute case numbers surged by 38% globally due to population aging (4).

In China, where adults aged 60 years and above now constitute 22% of the population (5), STI epidemiology in older demographics is currently presenting a critical yet understudied challenge. Previous research predominantly focused on HIV (6–11) and syphilis (12, 13), creating a striking knowledge gap regarding bacterial STI, particularly gonorrhoea caused by *Neisseria gonorrhoeae*, which ranks as the second most prevalent notifiable STI in China. Although sporadic reports indicated rising STI diagnoses among Chinese older adults, no longitudinal analysis has systematically examined gonorrhoea trends in this vulnerable group.

Gonorrhoea warrants particular concern due to its dual clinical impact, including increasing reproductive tract complications and growing antimicrobial resistance (14, 15). National surveillance data from 2018 to 2023 revealed a non-significant decline in overall incidence rate of gonorrhoea, masking potential age-specific epidemiological shifts (16). In this study, we aimed to delineate gonorrhoea incidence trends among Chinese older adults aged 60 years and above from 2004 to 2023, and develop an optimized Long Short-Term Memory (LSTM) model to forecast infection trajectories in the next 5 years. We anticipate that our study will serve as a valuable supplement to existing research on gonorrhoea and provide a meaningful reference for public health initiatives targeting older adults.

## 2 Data and methods

### 2.1 Data acquisition and categorization

Gonorrhoea surveillance data from 2004 to 2023 for older adults aged 60 years and above were extracted from China’s National Notifiable Disease Reporting System (NNDRS) managed by National Center for STD Control, the China CDC. Demographic denominators were obtained from the National Bureau of Statistics Yearbooks^1^. The study cohort was stratified using a two-dimensional framework (Supplementary Table S1) as follows. (1) Legal age subgroups aligned with China’s older adults rights protection law: Young-old (60–69 years), Middle-old (70–79 years), and Oldest-old (≥80 years); (2) sex-specific stratification: Male, female, and combined genders. The standardized annual incidence rates were indicated as cases per 100,000 population. Then, a total of 12 mutually exclusive categories were set up, enabling age-group and gender interaction analysis: male & 60–69 yrs, male & 70–79 yrs, male & 80 yrs and above, male & 60 yrs and above, female & 60–69 yrs, female & 70–79 yrs, female & 80 yrs and above, female & 60 yrs and above, male & female & 60–69 yrs, male & female & 70–79 yrs, male & female & 80 yrs and above, and male & female & 60 yrs and above.

### 2.2 Statistical analysis

#### 2.2.1 Temporal trend analysis

Joinpoint regression (v5.3.0; the US NCI Surveillance Research Program)^2^ was implemented to identify inflection points in incidence trends. Key parameters included: maximum allowed joinpoints (5, based on 20-year observation window), permutation test significance level (*α* = 0.05), annual percent change (APC) using weighted least squares, and model selection via Bayesian information criterion. The Monte Carlo permutation method (10,000 iterations) determined optimal segment configurations, with average APC (AAPC) computed across the entire study period.

#### 2.2.2 Deep learning forecasting

The construction of a LSTM predictive model was carried out utilizing the ‘keras’ package in R, with all training and testing procedures executed using R software version 4.2.1^3^. To enhance the accuracy of predictions, three distinct variants of the LSTM model were developed, including classic LSTM, stateful LSTM and stacked stateful LSTM (17). LSTM consisted of three gates for working as follows (18):the forget gate which could be expressed as


$$f_{t}=\sigma [w_{f}*(h_{t-1},x_{t})+b_{f}],$$

the input gate which could be expressed as


$$i_{t}=\sigma (w_{i}*[h_{t-1},x_{t}]+b_{i}),$$

$$\tilde{c_{t}}=\tanh(w_{c}*[h_{t-1},x_{t}]+b_{c}),$$

$$c_{t}=f_{t}*c_{t-1}+i_{t}*\tilde{c_{t}},$$

and the output gate which could be expressed as


$$o_{t}=\mathrm{\sigma c}w_{0}*[h_{t-1},x_{t}]+b_{0},$$

$$\;h_{t}=o_{t}*\tanh(c_{t}).$$

In the forget gate, f_t_, x_t_ were input and h_t_ were output vectors. In the input gate, vector C_t_ represented the cell state. In the output gate, vector O_t_, W and b represented the matrices of parameter. Optimized key parameters, including training sample percentage (70% or 80% of the total data set), number of dense units (ranging from 1 to 30) and layer units (ranging from 1 to 30), were tested and selected for downstream prediction with the least Root Mean Square Error (RMSE) during testing as description (18):


$$\;\text{RMSE}=\sqrt{\frac{1}{n}\sum_{t=1}^{n}{\left(\mathrm{yt}-y\right)}^{2}}$$

Where yt was the predicted value and y was the actual value, n denoted sample size.

For three variants of LSTM models, the one exhibiting superior performance was chosen for subsequent predictive analysis. By applying the optimized LSTM model, we trained on data spanning 20 years and predicted the value for the following year. Subsequently, we incorporated these predicted values into a sliding window to forecast the subsequent year’s outcomes. With the sliding window advancing annually, we were able to predict the five-year incidence rate of gonorrhoea.

## 3 Results

### 3.1 Temporal dynamics of gonorrhoea incidence rate (2004–2023)

**Figure 1:**
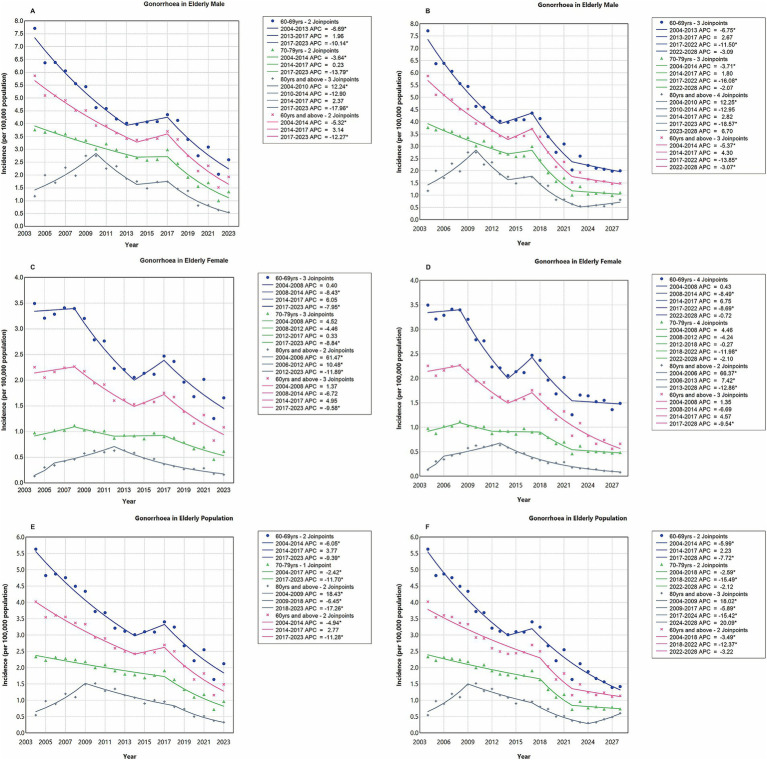
Temporal trend analysis of gonorrhoea incidence rates among older adults using joinpoint regression, stratified by gender and age group. Panels **A**, **C**, and **E** cover the period from 2004 to 2023, while Panels **B**, **D**, and **F** span from 2004 to 2028. Blue lines and icons represent the age group of 60 to 69 years; green lines and icons denote the age group of 70 to 79 years; gray lines and icons indicate individuals aged 80 years and older; pink lines and icons encompass all individuals aged 60 years and older. An asterisk (*) signifies statistical significance (*p* < 0.05).

In summary, the overall incidence rate of gonorrhoea among Chinese older adults (aged ≥60 years) exhibited a fluctuating decline trend with the AAPC of −5.84 (95% CI, −10.13 to −1.34). This nonlinear temporal trajectory was characterized by three distinct phases (Figure 1E; Supplementary Table S1). Phase I (2004–2014) had a sustained decline from 4.0205 to 2.4240 per 100,000 (APC, −4.94; 95% CI, −7.15 to −2.68). Phase II (2014–2017) had a non-significant rebound to 2.6957 per 100,000 (APC, 2.77; 95% CI, −24.05 to 39.07). And Phase III (2017–2023) had an accelerated reduction to 1.4897 per 100,000 (APC, −11.28; 95% CI, −15.70 to −6.63). The age-stratified analysis revealed that the incidence rate was higher in the younger age group with inverse correlations between age group and decline magnitude (Figure 1E). The overall magnitude of the decline in gonorrhoea incidence rate was relatively less pronounced among individuals aged 70–79 years (AAPC, −5.45; 95% CI, −7.03 to −3.84) and those aged ≥80 years (AAPC, −3.63; 95% CI, −6.82 to −0.33), as compared with the individuals aged 60–69 years (AAPC, −5.65; 95% CI, −10.00 to −1.09) (Figure 1E). The gender-specific analysis revealed that the incidence rate was consistently higher among older males compared with older females across all age groups (Figures 1A,C). In addition, the decline in the overall incidence rate was relatively less pronounced among older females (AAPC, −4.24; 95% CI, −9.95 to 1.84) compared with older males (AAPC, −6.31; 95% CI, −10.44 to −2.00) (Figures 1A,C). All ACP and AAPC values, together with their corresponding 95% CI, were provided in Supplementary Table S2.

### 3.2 LSTM model development and validation

**Figure 2:**
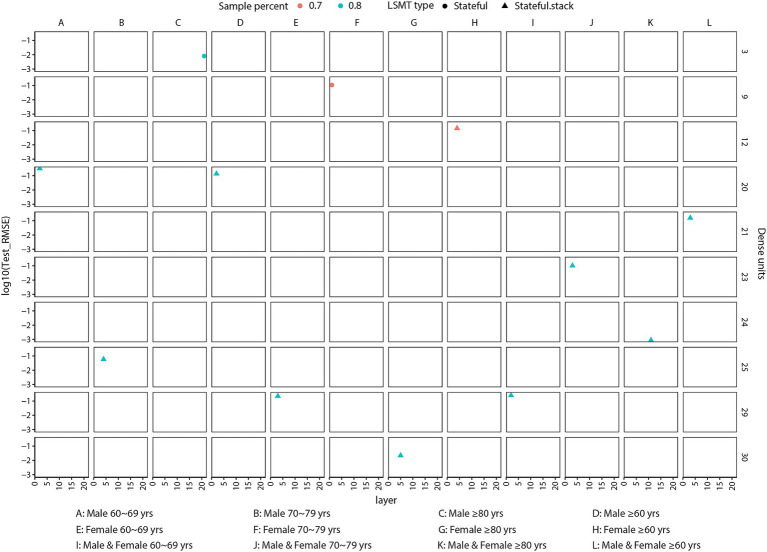
Optimized parameters for variants of long short-term memory model in predicting gonorrhoea incidence rates using a 20-year data-set. The sample percent indicates the proportion of samples used for training (either 70% or 80% of the data-set). The x-axis represents the number of layer units (ranging from 1 to 30). The right y-axis denotes the number of dense units (ranging from 1 to 30), while the left y-axis denotes the logarithmic transformation of the RMSE for predictions on the testing set. These parameters were selected to achieve the least RMSE for predicting the 12 available categories **(A–L)** using the LSTM models.

**Figure 3:**
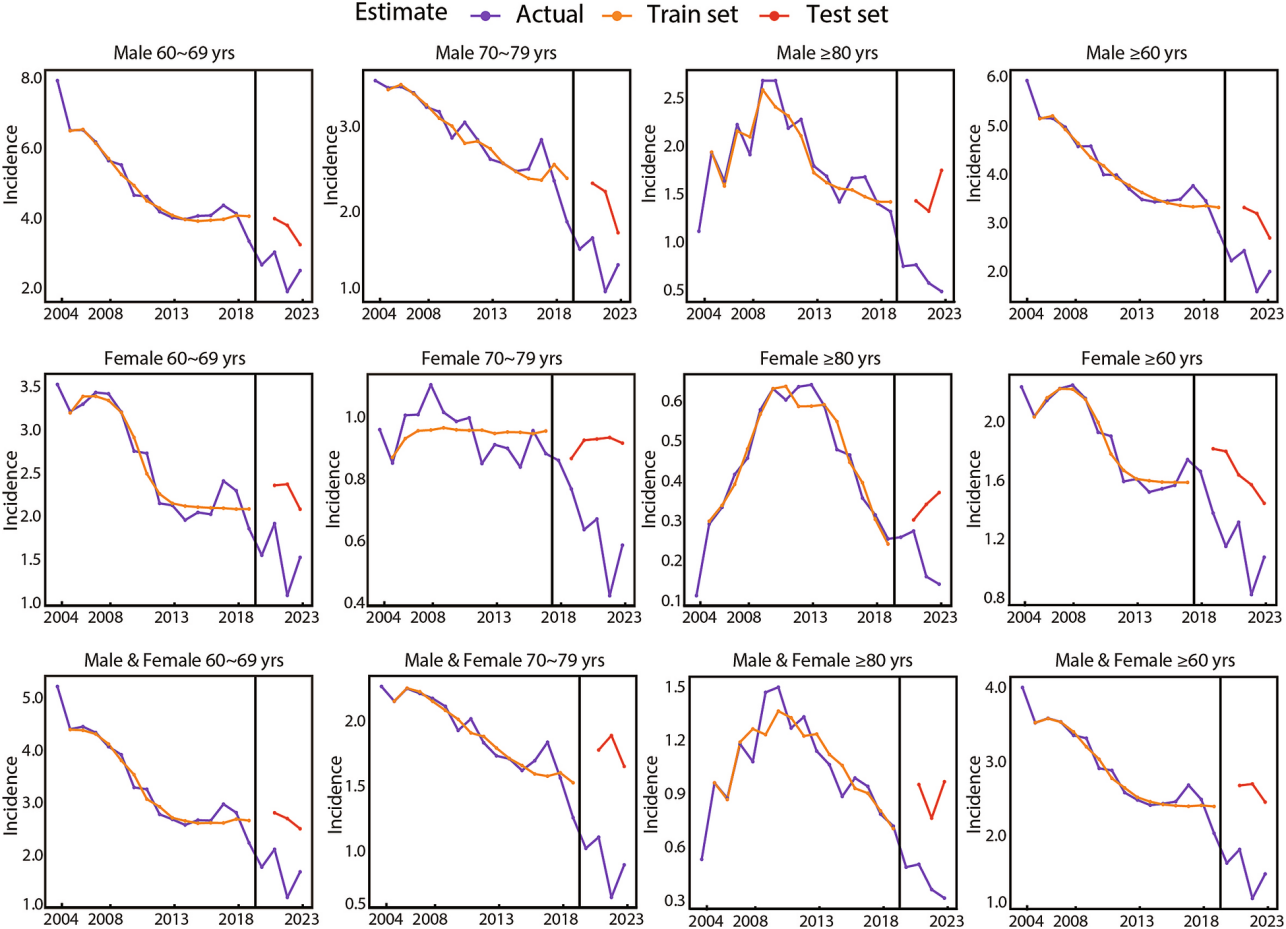
Presentation of actual values, training, and testing results using the optimized long short-term memory model, stratified by gender and age group.

Rather than arbitrarily setting parameters, we conducted a systematic evaluation of various combinations of layer units (ranging from 1 to 30), dense units (ranging from 1 to 30), and training sample sizes (70% or 80% of the total data-set) across three LSTM model configurations, including classic LSTM, stateful LSTM, and stacked stateful LSTM. This iterative process aimed to identify the parameter combination that yielded the lowest RMSE on the testing set, with each iteration running for 1,000 epochs (Supplementary Table S3). Based on these evaluations, the optimized parameters were selected as illustrated in Figure 2 and Supplementary Table S4. Notably, the classic LSTM model did not exhibit significant advantages over stateful or stacked stateful LSTM model across all 12 categories, with the latter two consistently achieving the lowest RMSE values (Figure 2; Supplementary Table S4). With optimized parameters, the LSTM models demonstrated precision and reliability in capturing trends within the training set. Although some discrepancies were observed in the testing set, likely attributable to the impact of the COVID-19 pandemic, the overall trend remained consistent with the actual data (Figure 3).

### 3.3 Five-year projections of gonorrhoea incidence rate (2024–2028)

**Figure 4:**
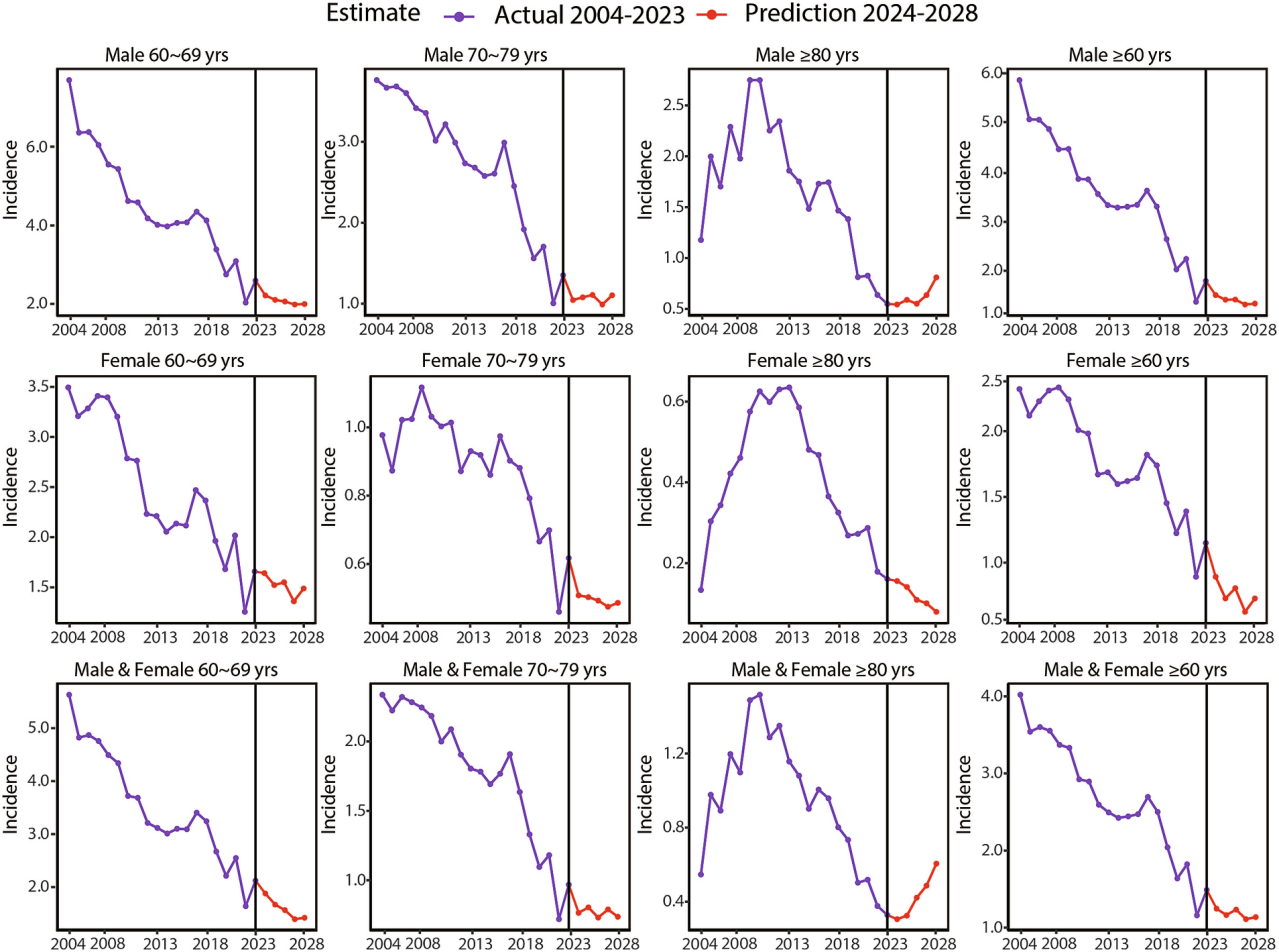
Projections of gonorrhoea incidence rates for the next 5 years using the optimized long short-term memory model, stratified by gender and age group.

The trend chart of gonorrhoea incidence rates stratified by gender and age group from 2004 to 2028 was presented in Figure 4. This included projected data for the years 2024 to 2028. Detailed forecasting methodologies and data, covering both the overall incidence rates and those stratified by gender and age group, were provided in Supplementary Figure S1 and Supplementary Table S5. The optimized LSTM model projected a stabilization trend in the overall incidence rates of gonorrhoea from 2022 to 2028 (APC, −3.22; 95% CI, −6.69 to 0.37; *p* = 0.075) (Figure 1F). Specifically, the projected incidence rates per 100,000 population were as follows: 1.2467 in 2024, 1.1637 in 2025, 1.2341 in 2026, 1.1103 in 2027, and 1.1373 in 2028. Notably, a pronounced upward trend was observed in the vulnerable population aged 80 years and above, with an estimated APC from 2024 to 2028 projected at 20.09 (95% CI, 7.71 to 33.89, *p* = 0.002) (Figure 1F). The model projections highlighted persistent gender disparities in the incidence rate of gonorrhoea across all age groups, with older males consistently exhibiting significantly higher rates compared with older females (Figures 1B,D). Specifically, the projected gender-specific incidence rates per 100,000 population for the years 2024 to 2028 were as follows: for males, the rates were 1.6510 in 2024, 1.5606 in 2025, 1.5634 in 2026, 1.4666 in 2027, and 1.4839 in 2028; for females, the rates were 0.8260 in 2024, 0.6619 in 2025, 0.7396 in 2026, 0.5596 in 2027, and 0.6613 in 2028.

## 4 Discussion

The 21st century has witnessed unprecedented demographic transformations, with China’s population aged 60 years and above projected to exceed 400 million by 2035, surpassing the current total population of the United States (19–21). This significant demographic shift necessitates immediate and comprehensive analysis of its diverse societal impacts, including sexual health among older adults. Many older adults continue to remain sexually active well into their later years. However, various biologic, behavioral, and social factors may contribute to an increased risk of STI. These factors include the use of erectile dysfunction medications or aphrodisiacs, compromised immune function, participation in low-cost commercial sex, and infrequent condom use (22). Additionally, inadequate sexual health awareness, stigma, and shame contribute to lower rates of STI testing, diagnosis, and treatment, thereby facilitating the transmission of STI and accelerating disease progression within this population (23, 24).

A global burden of disease study on STI among individuals aged 60 to 89 years demonstrated that, despite significant progress in reducing HIV infection rates, the age-standardized incidence of other STI remained relatively unchanged from 1990 to 2019 (4). According to surveillance data from the CDC Atlas Plus system, the United States witnessed a fivefold increase in rates of syphilis and more than a twofold increase in rates of gonorrhoea and chlamydia among individuals aged 65 years and older between 2009 and 2019 (25). Nevertheless, geriatric sexual health remains systematically marginalized in both clinical practice and public health frameworks, resulting in substantial underestimation of disease burden (25, 26). This oversight is particularly concerning given China’s accelerated aging process, where existing studies among Chinese older adults predominantly focused on HIV and syphilis (6, 7, 11–13), leaving critical knowledge gaps regarding other STI.

Gonorrhoea is the second most prevalent bacterial STI globally, posing substantial public health challenges due to its relatively high incidence and escalating antibiotic resistance (14, 27). This condition can affect multiple anatomical sites including the urogenital tract, oropharynx, rectum, and conjunctiva (15). Recurrent infections are common as there are no vaccines available and host immunity does not confer protection against reinfection (14, 15). The presence of gonorrhoea also increases the risk of acquiring and transmitting HIV. According to World Health Organization (WHO) estimates, in 2020 there were approximately 82.4 million new cases of gonorrhoea among adults aged 15 to 49 years (28). Currently, the WHO does not provide specific epidemiological data regarding gonorrhoea infections in older adults globally.

Our comprehensive analysis of Chinese disease surveillance data from 2004 to 2023 identified three key findings. First, the incidence rates of gonorrhoea among older adults aged 60 years and above exhibited a generally declining yet fluctuating trend, with a consistent gender disparity favoring male predominance across all age groups. Second, age-stratified analysis indicated that younger age groups had higher incidence rates, with an inverse relationship observed between age groups and the magnitude of rate decline. Third, there was a notable decline in incidence rates of gonorrhoea from 2020 to 2023, likely attributable to the disruptions caused by the COVID-19 pandemic. These disruptions included decreased sexual activities, reduced medical attention for individuals with mild symptoms, and interruptions in surveillance activities (29, 30). Theoretically, in the absence of the COVID-19 pandemic, the incidence rates of gonorrhoea among older adults in China would have shown a stable trend from 2020 to 2023. This projection was supported by the data observed in the testing set during the LSTM modeling process, as illustrated in Figure 3.

When constructing the prediction model, the LSTM model was ultimately chosen due to its demonstrated superior performance in handling time series data (31, 32). In recent years, LSTM models have progressively exhibited enhanced predictive capabilities in the analysis of infectious diseases (33, 34). Specifically, in the context of predicting STI, Zhu and colleagues compared several approaches and found that LSTM models outperformed other models (35). In this study, the optimized LSTM model predicted that overall incidence rates of gonorrhoea would stabilize over the next 5 years following the COVID-19 pandemic. This contrasted with the rising trends in STI among older adults observed in other regions, including the United States and England (4, 26, 36, 37), indicating the partial effectiveness of China’s current control measures. In the forthcoming years, the availability of more sensitive diagnostic tools may identify additional asymptomatic cases, while shifts in sexual behavior, influenced by apps that facilitate partner identification, are likely to lead to an increase in new infections. Consequently, it is important to note that actual incidence rates may exceed currently predicted values. Therefore, achieving WHO’s 2030 reduction target for gonorrhoea, which aims for a 90% decrease from the 2018 baseline, will necessitate intensified and comprehensive interventions (38).

Based on the above results, we propose a multidimensional intervention framework that incorporates several key strategies. Firstly, integrating routine STI screening into geriatric care protocols is essential to ensure timely diagnosis and effective management for older adults. Secondly, age-appropriate sexual health education programs can empower older adults to adopt safer sexual practices. Furthermore, clinician training should focus on enhancing awareness and sensitivity to manifestations of STI in older adults, as well as providing stigma-free counseling. Finally, increased STI monitoring, along with a more comprehensive understanding of sexual networks and health-seeking behaviors, will contribute to a better grasp of the risk factors for STI among older adults. These strategies will facilitate the reduction of the STI burden and promote healthy aging in this demographic.

As a national-level epidemiological study on gonorrhoea among older adults in China, our findings contribute to evidence-based strategies for the Healthy Aging 2030 initiatives and enhance the global understanding of gonorrhoea burden. However, several limitations should be acknowledged. Firstly, the absence of anatomical site data hindered in-depth analyses of sexual behavior patterns. Secondly, it was not feasible to stratify the study population based on sexual behavior patterns. For instance, among older men who have sex with men, the incidence of gonorrhoea might increase rather than decrease. These factors are essential for refining future prevention and control strategies. Thirdly, despite the presence of regional economic heterogeneity, geographic stratification was not available in the existing surveillance data. Fourthly, the reliance on passive surveillance systems may have led to potential under-reporting of cases, resulting to underestimation of disease burden. Lastly, the COVID-19 pandemic may have influenced incidence forecasting. To address these gaps, future research should incorporate anatomical site data, key high-risk population, geographic stratification, more active surveillance system and mixed-methods behavioral studies.

## 5 Conclusion

In China, gonorrhoea remains a significant STI. Healthcare providers should recognize that, given the aging population, the risk of gonococcal infection among older adults may be increasing, potentially leading to a rise in the absolute number of cases. Therefore, it is crucial to develop interventions that are appropriate for this age group to effectively reduce incidence rates and prevent transmission to sexual partners.

## Data Availability

The original contributions presented in the study are included in the article/Supplementary material, further inquiries can be directed to the corresponding authors.

## Footnotes

^1^ https://www.stats.gov.cn/english/

^2^ https://surveillance.cancer.gov/joinpoint/download

^3^ https://www.r-project.org/
