# Supplementary figures and images for: Gonorrhoea among China’s aging population: a 20-year nationwide analysis of epidemiological trends with 5-year projections
Source: Front Public Health. 2025 Jun 27;13:1594289. doi: 10.3389/fpubh.2025.1594289 (PMC12245903; doi:10.3389/fpubh.2025.1594289)

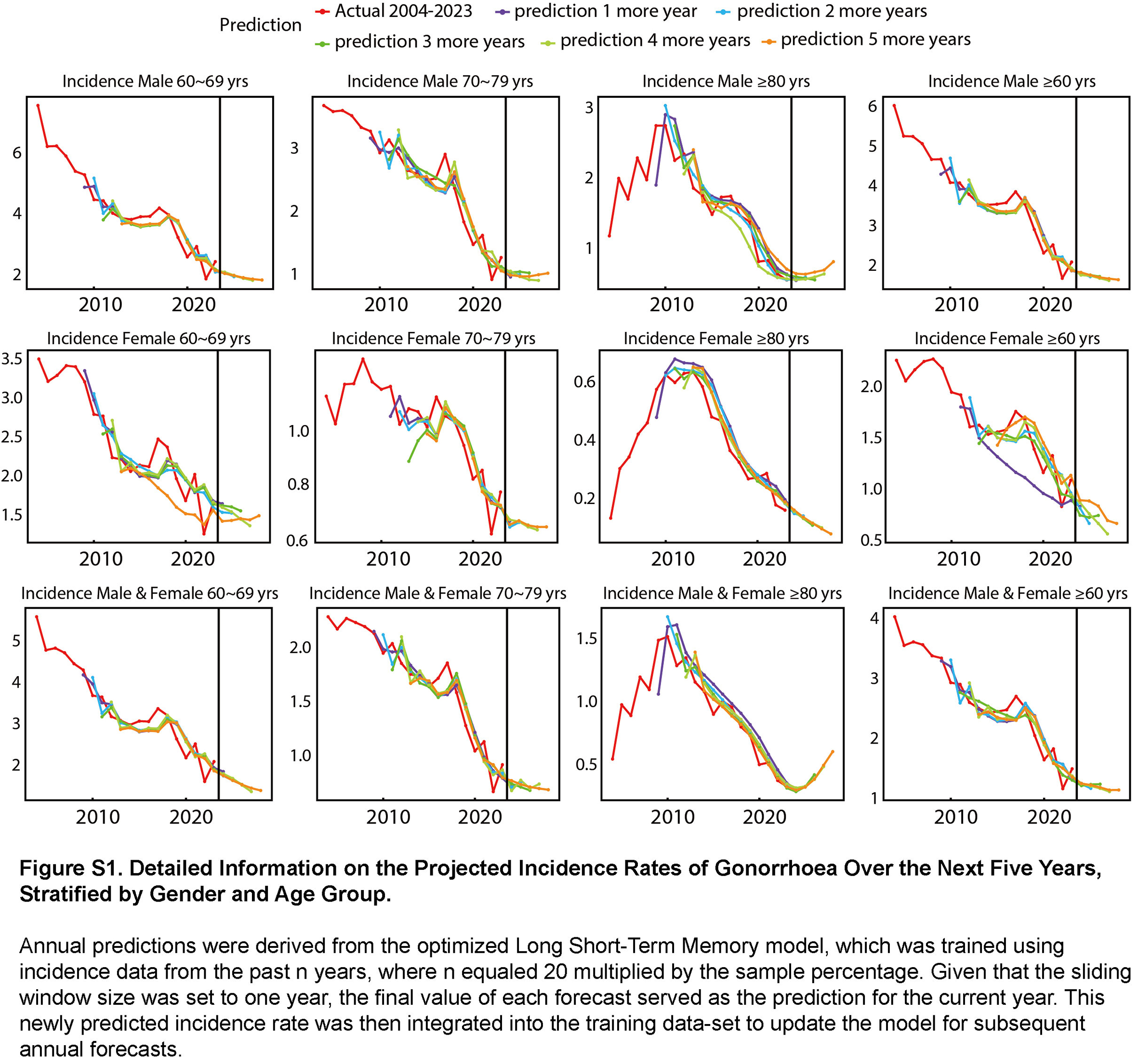

Supplement: Supplementary file 1 [file Image_1.JPEG]
