# Supplementary material for: Gonorrhoea among China’s aging population: a 20-year nationwide analysis of epidemiological trends with 5-year projections
Source: Front Public Health. 2025 Jun 27;13:1594289. doi: 10.3389/fpubh.2025.1594289 (PMC12245903; doi:10.3389/fpubh.2025.1594289)
Supplement: Supplementary file 2 [file Table_1.DOCX]

| Year |  | Male | | | |  | Female | | | |  | Male and Female | | | |  |
| --- | --- | --- | --- | --- | --- | --- | --- | --- | --- | --- | --- | --- | --- | --- | --- | --- |
|  |  | 60~69yrs | 70~79yrs | ≥80yrs | Total |  | 60~69yrs | 70~79yrs | ≥80yrs | Total |  | 60~69yrs | 70~79yrs | ≥80yrs | Total | |
| 2004 |  | **3543 (7.7126)** | **982 (3.7634)** | **75 (1.1775)** | **4600 (5.8673)** |  | **1566 (3.4949)** | **268 (0.9773)** | **13 (0.1337)** | **1847 (2.2538)** |  | **5109 (5.6300)** | **1250 (2.3357)** | **88 (0.5469)** | **6447 (4.0205)** | |
| 2005 |  | **3023 (6.3712)** | **996 (3.6688)** | **139 (1.9975)** | **4158 (5.0984)** |  | **1462 (3.2087)** | **254 (0.8732)** | **32 (0.3037)** | **1748 (2.0519)** |  | **4485 (4.8220)** | **1250 (2.2228)** | **171 (0.9773)** | **5906 (3.5419)** | |
| 2006 |  | **3197 (6.3860)** | **1056 (3.6849)** | **124 (1.7025)** | **4377 (5.0893)** |  | **1575 (3.2859)** | **308 (1.0221)** | **37 (0.3435)** | **1920 (2.1612)** |  | **4772 (4.8696)** | **1364 (2.3201)** | **161 (0.8917)** | **6297 (3.6015)** | |
| 2007 |  | **3112 (6.0566)** | **1059 (3.6027)** | **181 (2.2886)** | **4352 (4.9072)** |  | **1690 (3.4100)** | **316 (1.0243)** | **47 (0.4219)** | **2053 (2.2425)** |  | **4802 (4.7572)** | **1375 (2.2823)** | **228 (1.1971)** | **6405 (3.5537)** | |
| 2008 |  | **2920 (5.5579)** | **1044 (3.4170)** | **168 (1.9769)** | **4132 (4.5114)** |  | **1734 (3.3960)** | **355 (1.1170)** | **54 (0.4606)** | **2143 (2.2661)** |  | **4654 (4.4925)** | **1399 (2.2442)** | **222 (1.0978)** | **6275 (3.3709)** | |
| 2009 |  | **3031 (5.4418)** | **1048 (3.3568)** | **239 (2.7479)** | **4318 (4.5160)** |  | **1731 (3.2030)** | **328 (1.0313)** | **69 (0.5752)** | **2128 (2.1749)** |  | **4762 (4.3393)** | **1376 (2.1833)** | **308 (1.4885)** | **6446 (3.3320)** | |
| 2010 |  | **2577 (4.6267)** | **941 (3.0141)** | **239 (2.7479)** | **3757 (3.9292)** |  | **1506 (2.7867)** | **319 (1.0030)** | **75 (0.6252)** | **1900 (1.9419)** |  | **4083 (3.7206)** | **1260 (1.9992)** | **314 (1.5175)** | **5657 (2.9241)** | |
| 2011 |  | **2417 (4.5907)** | **922 (3.2181)** | **204 (2.2525)** | **3543 (3.9211)** |  | **1431 (2.7638)** | **306 (1.0141)** | **76 (0.5988)** | **1813 (1.9156)** |  | **3848 (3.6849)** | **1228 (2.0876)** | **280 (1.2874)** | **5356 (2.8952)** | |
| 2012 |  | **2349 (4.1839)** | **868 (2.9910)** | **221 (2.3434)** | **3438 (3.6344)** |  | **1245 (2.2321)** | **266 (0.8716)** | **82 (0.6302)** | **1593 (1.6042)** |  | **3594 (3.2112)** | **1134 (1.9047)** | **303 (1.3502)** | **5031 (2.5947)** | |
| 2013 |  | **2386 (4.0185)** | **792 (2.7352)** | **192 (1.8578)** | **3370 (3.4155)** |  | **1309 (2.2111)** | **287 (0.9304)** | **88 (0.6349)** | **1684 (1.6207)** |  | **3695 (3.1161)** | **1079 (1.8043)** | **280 (1.1573)** | **5054 (2.4949)** | |
| 2014 |  | **2485 (3.9776)** | **806 (2.6816)** | **190 (1.7513)** | **3481 (3.3672)** |  | **1299 (2.0549)** | **288 (0.9191)** | **86 (0.5853)** | **1673 (1.5314)** |  | **3784 (3.0106)** | **1094 (1.7820)** | **276 (1.0805)** | **5154 (2.4240)** | |
| 2015 |  | **2703 (4.0678)** | **785 (2.5782)** | **163 (1.4831)** | **3651 (3.3841)** |  | **1422 (2.1359)** | **279 (0.8608)** | **73 (0.4808)** | **1774 (1.5538)** |  | **4125 (3.1009)** | **1064 (1.6926)** | **236 (0.9017)** | **5452 (2.4431)** | |
| 2016 |  | **2812 (4.0800)** | **819 (2.6076)** | **203 (1.7297)** | **3834 (3.4212)** |  | **1478 (2.1160)** | **324 (0.9742)** | **74 (0.4679)** | **1876 (1.5775)** |  | **4290 (3.0915)** | **1143 (1.7675)** | **277 (1.0053)** | **5710 (2.4720)** | |
| 2017 |  | **3133 (4.3529)** | **964 (2.9903)** | **216 (1.7434)** | **4313 (3.6989)** |  | **1807 (2.4698)** | **313 (0.9025)** | **60 (0.3655)** | **2180 (1.7544)** |  | **4940 (3.4036)** | **1277 (1.9082)** | **276 (0.9583)** | **6493 (2.6957)** | |
| 2018 |  | **3075 (4.1291)** | **827 (2.4526)** | **181 (1.4662)** | **4083 (3.3874)** |  | **1782 (2.3665)** | **322 (0.8813)** | **56 (0.3254)** | **2160 (1.6738)** |  | **4857 (3.2429)** | **1149 (1.6355)** | **237 (0.8019)** | **6243 (2.5014)** | |
| 2019 |  | **2495 (3.3884)** | **685 (1.9179)** | **177 (1.3828)** | **3357 (2.7482)** |  | **1469 (1.9633)** | **309 (0.7924)** | **48 (0.2686)** | **1826 (1.3866)** |  | **3964 (2.6701)** | **994 (1.3305)** | **225 (0.7337)** | **5183 (2.0418)** | |
| 2020 |  | **2014 (2.7510)** | **607 (1.5598)** | **124 (0.8127)** | **2745 (2.1549)** |  | **1247 (1.6811)** | **279 (0.6657)** | **56 (0.2726)** | **1582 (1.1578)** |  | **3261 (2.2125)** | **886 (1.0961)** | **180 (0.5028)** | **4327 (1.6389)** | |
| 2021 |  | **2200 (3.0908)** | **704 (1.7043)** | **134 (0.8268)** | **3038 (2.3606)** |  | **1464 (2.0170)** | **313 (0.6988)** | **62 (0.2873)** | **1839 (1.3234)** |  | **3664 (2.5487)** | **1017 (1.1812)** | **196 (0.5187)** | **4877 (1.8222)** | |
| 2022 |  | **1493 (2.0322)** | **445 (1.0047)** | **108 (0.6377)** | **2046 (1.5190)** |  | **942 (1.2552)** | **222 (0.4597)** | **40 (0.1789)** | **1204 (0.8264)** |  | **2435 (1.6396)** | **667 (0.7205)** | **148 (0.3766)** | **3250 (1.1591)** | |
| 2023 |  | **1907 (2.5957)** | **599 (1.3524)** | **93 (0.5491)** | **2599 (1.9296)** |  | **1244 (1.6577)** | **298 (0.6171)** | **36 (0.1610)** | **1578 (1.0831)** |  | **3151 (2.1217)** | **897 (0.9689)** | **129 (0.3283)** | **4177 (1.4897)** | |

**Table S1. Annual Cases and Incidence Rates of Gonorrhoea per 100,000 Population, Stratified by Gender and Age Group.**
